# Marine nature conservation and conflicts with fisheries

**DOI:** 10.1007/s13280-019-01279-7

**Published:** 2019-11-20

**Authors:** Kjell Grip, Sven Blomqvist

**Affiliations:** 1 Department of Ecology, Environment and Plant Sciences, Stockholm University, 106 91 Stockholm, Sweden; 2 Present Address: Mandelblomsgatan 11, 745 36 Enköping, Sweden

**Keywords:** Ecosystem-based management, Fishery, Marine nature conservation, Marine protected area, Marine spatial planning

## Abstract

Globally, conflicts between marine nature conservation and fishery interests are common and increasing, and there is often a glaring lack of dialogue between stakeholders representing these two interests. There is a need for a stronger and enforced coordination between fishing and conservation authorities when establishing marine protected areas for conservation purposes. We propose that an appropriate instrument for such coordination is a broad ecosystem-based marine spatial planning procedure, representing neither nature conservation nor fishery. Strategic environmental assessment for plans and programmes and environmental impact assessment for projects are commonly used tools for assessing the environmental impacts of different human activities, but are seldom used for evaluating the environmental effects of capture fisheries. The diversity of fisheries and the drastic effects of some fisheries on the environment are strong arguments for introducing these procedures as valuable supplements to existing fisheries assessment and management tools and able to provide relevant environmental information for an overall marine spatial planning process. Marine protected areas for nature conservation and for protection of fisheries have different objectives. Therefore, the legal procedure when establishing marine protected areas should depend on whether they are established for nature conservation purposes or as a fisheries resource management tool. Fishing in a marine protected area for conservation purpose should be regulated according to conservation law. Also, we argue that marine protected areas for conservation purposes, in the highest protection category, should primarily be established as fully protected marine national parks and marine reserves.

## Introduction

Worldwide, conservation conflicts with fishery interests are common. A recent example is the failure to create the large *Antarctic Ocean Sanctuary.* The Commission for the Conservation of Antarctic Marine Living Resources, in November 2018, could not agree on the establishment of the sanctuary. The delegations from China, Norway and Russia voted against the proposal to create three new marine protected areas (MPAs) to protect Antarctic marine living resources, in the Weddel Sea and in the Western Antarctic Peninsula, respectively (ASOC 2018). This is a clear example of the common global conflict between marine nature conservation and fishery interests.

Compared with terrestrial protected areas, few MPAs have been established. Most of these are small, situated in coastal areas, not properly enforced and commonly in conflict with the fishery (Barcott 2011; WRI 2011; Grip and Blomqvist 2018). Conservation and fishery interests are usually planned and managed apart, in strictly sectoral procedures by separate ministries, agencies and legal frameworks. Also, the two interests are related to different global and regional environmental and fishery organizations, which can be powerful political players (Redpath et al. 2013). This strict sectoral division of the activities complicates cooperation and coordination in a multisectoral marine spatial planning (MSP) and decision-making process, when MPAs are established for conservation purposes (Rossiter and Lopez-Carr 2016).

*Different categories of MPAs*

MPAs are tools for protecting and conserving the marine environments and its biodiversity, but are also used for fisheries resource management. According to the International Union for Conservation of Nature (IUCN), MPAs in different management categories should be classified according to their objectives (Dudley 2008; Laffoley et al. 2019). There are MPAs both for nature conservation (e.g. marine national parks and reserves) and for fishery purposes (e.g. no-take zones and sanctuaries), but they have different objectives, nature conservation and resource management, respectively. Our focus is on conflicts with the fishery when establishing MPAs in the strongest protection category, classified as marine national parks and marine reserves.

### Aim

The aim of this study is to review the common approach for handling conflicts between marine nature conservation and the fishery when establishing MPAs for conservation purposes and to propose how to strengthen this process. We argue:

(i) that the appropriate instrument for coordinating different marine interests is a broad ecosystem-based planning^1^ and a decision-making process that is not tied to the interest of a specific sector;
(ii) that strategic environmental assessment (SEA) (Dalal-Clayton and Sadler 2005) and environmental impact assessment (EIA) (Glasson et al. 2012) are tools that should be used in fisheries management, to provide relevant information on the environmental effects of particular fisheries and to supplement fisheries assessment tools in the MSP process;
(iii) that the legal procedure when establishing MPAs for nature conservation and for fishery purposes should be clearly separated under conservation and fishery law, respectively; and
(iv) that the establishment of MPAs for nature conservation purposes in the highest protection category should focus on fully protected marine national parks and marine reserves.

## Materials and methods

**Fig. 1:**
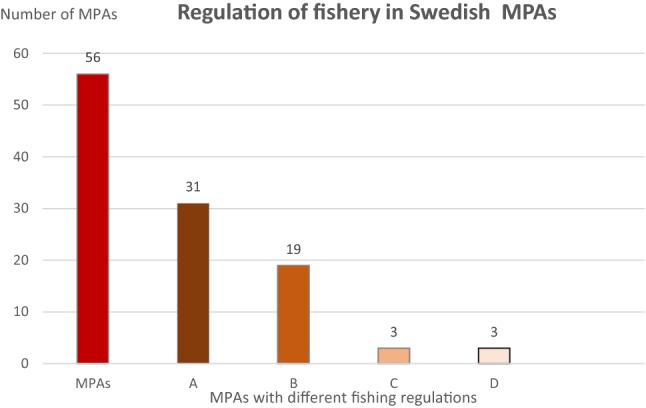
Regulation of fisheries in Swedish MPAs (marine nature reserves and national parks) according to different Swedish laws, 2015. Clear red = total number of MPAs; A = fishing not regulated; B = fishing regulated according to fisheries legislation; C = fishing regulated according to the environmental code; D = fishing forbidden according to the environmental code. Source: Public records and interviews with nature conservation officers at coastal CABs, based on data from (Grip and Blomqvist 2018)

The present study is based mainly on information gained from interviewing representatives of all 14 coastal County Administrative Boards (CABs) responsible for the establishment of MPAs in Sweden (Fig. 1). Our interviews focused on the often conflicting interests of marine nature conservation and fisheries resource management when MPAs are established for conservation purposes. Measures and tools that potentially can alleviate or overcome the conflicts are proposed.

The personal experiences of one of us (KG) of handling conservation conflicts with the fishery when establishing MPAs at the Swedish Environmental Protection Agency, the National Fishery Board (now closed) and in several international environmental organizations underlie our studies. In addition, we used various databases accessible through Internet and at university libraries.

Finally, our review looks ahead and emphasizes four aspects of action (a–d) worthy of closer consideration in managing conflicting interests, when establishing MPAs for conservation purposes.

## Marine nature conservation

Marine nature reserves are often established with the purpose to protect, restore or create valuable marine habitats or habitats for endangered species. Designation and establishment of MPAs for such conservation purposes often interferes with established fisheries and the livelihoods of fishing communities (Barcott 2011; WRI 2011). Fishing as a matter of course affects the food webs and ecosystems that you want to protect (Dayton et al. 1995).

Controversies between conservation and fisheries are most common in coastal waters at national level, but also occur regionally and globally concerning what should be protected in an MPA—the ecological values or the socio-economic value of the commercial fishery (Pita et al. 2011; Kearney et al. 2012; Laffoley et al. 2019).

The basis for the conflicts between the two interests is that their requirements for the same space are usually difficult to reconcile. The perceived degree of encroachment on the respective interest must be dealt with in negotiations between the parties involved. A nature conservation interest aims at protecting different habitats and species, while the commercial fishery wants to use the fish as a resource. The strength of the conflict depends on the strength of the encroachment and involved legislation.

The fishery is often backed up by strong sector legislation and strong user organizations. The conservation interest can also be supported by strong environmental organizations and strong conservation legislation, but these are usually weaker than on the fishery side. Of course, nature conservation and fishing interests can interact, but it is important to understand that the objectives of MPAs for conservation purposes and fishery resource management are different.

In our interviews of conservation officers regarding the management of marine nature reserves at all coastal CABs in Sweden, the need for better coordination between nature conservation and fishing interests was emphasized by the respondents (Fig. 1). Several argued that fishing in a marine nature reserve should be regulated according to the conservation law or, if under fishery law, in line with conservation law (Grip and Blomqvist 2018). It should be noted that Sweden’s as well as other countries’ experiences of how to handle fishing in MPAs is anchored in and influenced by the coordinated work of the regional marine environmental commissions (Box 1).

**Table:** 

**Box 1.** The regional marine environmental commissions and fishery	
The regional environmental commissions (e.g. HELCOM, OSPAR and UNEP Regional Seas Programme) can address the environmental effects of fishing, but not fisheries resource management, which is the responsibility of the fisheries authorities and organizations. Instead, in HELCOM, for example these issues are discussed within a separate forum the *Baltic Sea Fisheries Forum* (BALTFISH). Also, to assist the governments in managing fisheries in Baltic MPAs, HELCOM has developed a special project: Managing Fisheries in Baltic MPAs (BALTFIMPA).	

### Marine Protected Areas for nature conservation

MPAs have long been used as a conservation tool for the benefit of particular fisheries. Today, MPAs are subject to increasing policy attention and are more commonly seen as a nature conservation tool, intended primarily to protect and preserve various marine ecosystems and their biodiversity with the ecological services they provide (Hoagland et al. 2001; Laffoley et al. 2019; Maestroa et al. 2019). National MPA legislation varies widely between countries according to the form of government, public administrative structures and etcetera (Pomeroy et al. 2005; Barcott 2011). It has been argued that the establishment of MPAs for conservation purposes should focus on the strongest protection category (e.g. marine national parks and marine reserves) and be regulated in accordance with conservation law (Costello and Ballantine 2015; Costello 2018).

Fishing in an MPA established for nature conservation purposes may be unregulated, but is normally regulated in some way (FAO 2011), usually under fishery laws and jurisdiction. (Mesnildrey et al. 2013; Grip and Blomqvist 2018) (Fig. 1). Such regulation is normally based on negotiations between the two interested parties and depends on the prevailing political and institutional conditions and on law enforcement agencies that enforce the regulations. In the negotiations, the institutions usually aim at achieving a prosperous fishing with minimal impact on the environment. The outcome is usually a compromise, where the fishery is regulated to mitigate its effects on habitats and species. However, MPAs that allow fishing, although regulated, cannot protect all aspects of biodiversity.

Fishing that claims to be sustainable is allowed in more than 94% of all MPAs (Costello and Ballantine 2015). MPAs designated for conservation purposes or as fisheries resource management tools have different objectives and therefore their management should be kept legally separated under conservation and fishery jurisdiction, respectively (Hilborn 2016).

Management of MPAs according to two different laws, under different administrations, each eagerly defending its mandate, easily leads to target conflicts. Strictly protected conservation MPAs (no fishing allowed) according to conservation law, as, for example marine nature reserves in New Zealand, are not common (Enderby and Enderby 2006). The positive long-time experiences of strictly protected marine nature reserves in New Zealand, based on socio-economic and ecological factors, should be applicable worldwide and deserves to be tried widely (Ballantine 2014). Also, strictly protected areas have often proven to be beneficial to fishing outside the MPA (Costello 2018).

#### MPAs in the High Seas

MPA is also an instrument for protecting marine ecosystems and living resources in the High Seas, including the seabed. However, High Seas MPAs^2^ have yet to be incorporated formally into international law, e.g. as a treaty under United Nations Convention on the Law of the Sea (Houghton 2014; Anonymous 2018). The Regional Seas Conventions can designate MPAs in the High Seas, but such areas still lack legal protection. In 2010, as an example, a network of six high seas marine protected areas was designated by the OSPAR Commission in coordination with the North-East Atlantic Fisheries Commission (O’Leary et al. 2012).

## Fishery

Globally, fishing is an important commercial activity and fish is a high-quality protein source for billions of people (FAO 2016a). Fish and fishing are an integral part of most human societies and make important contributions to economic and social health and well-being in many countries and areas. The fishery exploits marine ecosystems, and many fishing practices have side effects on the bottom fauna and produce unwanted by-catches (Ackefors 2002).

Fishery resource management aims at balancing benefits in the form of food against adverse effects on marine ecosystems. The small-scale coastal fisheries and the large-scale industrial fisheries differ greatly, e.g. concerning regulations, gear-use and sustainability (Guyader et al. 2013). Also, the small-scale fishing is closer to the local business community, as shown, e.g. by the EU Leader project on Sustainable fishery in the Sound (www.skaansomtkystfiskeri.dk).

*Sustainability in fisheries*

The FAO Code of Conduct for responsible fisheries complemented by its Technical Guidelines is the foundation for assisting states in building good management practices for sustainable fisheries in the future (FAO 2011). Sustainability in fisheries is about continuing in the long term to produce the benefits to society that natural systems provide (FAO 2016b). Unsustainable fishing can affect both target and nontarget species and their habitats, particularly if there are lasting ecological impacts, such as habitat destruction from bottom trawling (Costello 2018). Even sustainable fishing alters ecosystems by removing certain life stages of species and can thus affect the species you want to protect in a conservation MPA (Box 2).

**Table:** 

**Box 2.** Promoting sustainable fishery	
Sustainability in fishery is addressed in Goal 14, *Conserve and sustainably use the oceans, seas and marine resources (Life below water)*, of the Sustainable Development Goals of Agenda 2030. As reflected in this goal, there is an increased focus on the contribution of fisheries towards food security, nutrition and sustainable economic growth. The Marine Stewardship Council (MSC) is one of several non-governmental organizations working to promote sustainability in the use of marine resources, advancing ocean stewardship and protecting habitats. MSC has provided seafood buyers and consumers with a mean to use their purchasing power to promote more sustainable practices in the fishing industry. The work of MSC contributes to the protection of species and habitats and to ease the conflicts between fishery and marine nature conservation (MSC 2017). However, as mentioned above, even sustainable fisheries affect the species and habitats you want to protect in a conservation MPA. It should be noted that sustainable fishery means, for example that the effect on ecosystems is limited, that the recruitment is not threatened and that a good fisheries governance is in place.	

Certain capture fisheries presently undergo rapid socio-ecological changes, related to changing ecosystems and levels of fish extraction (Campling et al. 2018). For example, the new (2014) EU Common Fisheries Policy (COM (2011) 425) was intended to promote good maritime governance and responsible fishing worldwide. This is in line with the European Maritime Policy (EU/EC 2007) promoting an ecosystem-based marine spatial planning, and a more open and coherent decision-making process, taking other policy areas into account. In fisheries today, there is an increased focus on resource and ecosystem sustainability and use of stock management policies. Also, policies and practices for preventing illegal, unreported and unregulated fishing have been revised in line with internationally recognized best policies and practices (Delpeuch and Hutniczak 2019). However, these approaches have not yet been recognized by all national governments and implemented in their legislation and management practices.

### MPAs for fisheries resource management

Fishing is a strong driver of environmental changes, leading to increasing demands for conservation (Worm et al. 2009; Rochet et al. 2010). Interest in the establishment of MPAs for fisheries resource management under fishery law, e.g. time-limited marine no-take areas and marine sanctuaries for spawning and migration purposes, has increased in recent years (Ward and Hegerl 2003; Hoffmann and Pérez-Ruzafa 2009; Krueck et al. 2017). The reason is the depletion of many fish stocks and the continued decline in many marine fish resources. This decline needs to be reversed, i.e. by reducing fishing pressure and establishing areas permanently or temporally closed for fishing.

The purpose of closed areas or no-take MPAs in fisheries legislation is generally to prevent the capture of juvenile fish, to protect spawning aggregations or to protect other sensitive species and habitats from the adverse effects of fishing. For a no-take MPA to be considered a fisheries management tool, the objective, sustained or increased yield, needs to be clearly stated, and distinguished from other possible conservation objectives (Pendleton et al. 2017). It should be noted that no-take MPAs for fishery purposes are unlikely to protect fish stocks that are primarily highly mobile (Habtes 2014; Krueck et al. 2017).

**Fig. 2:**
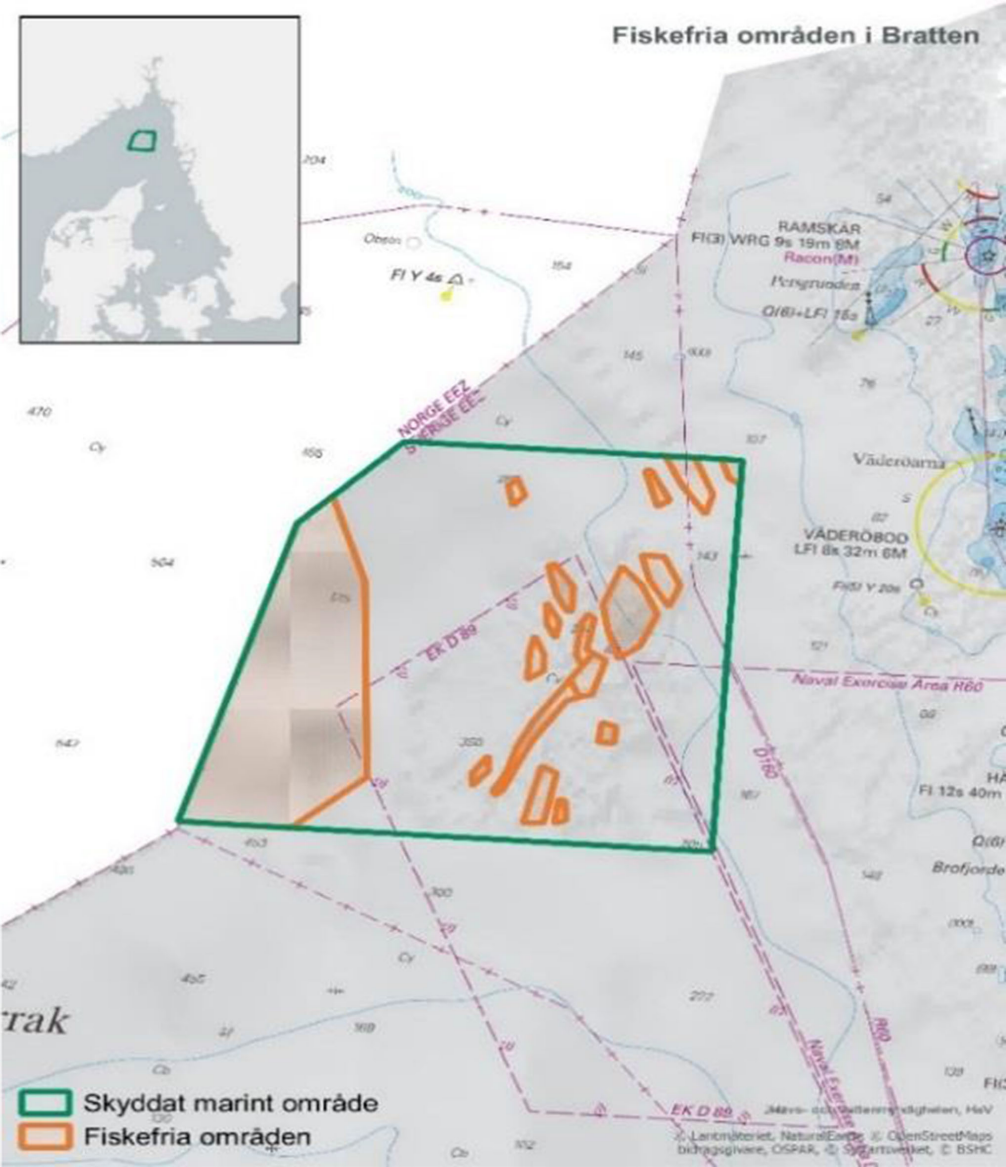
Nautical chart with examples of regulated fishing in Bratten Marine Protected Area (Natura 2000) in Skagerrak. Green: MPA (Natura 2000) according to Swedish jurisdiction. Orange: areas where fishing is not allowed—no-take zones—according to EU’s fisheries jurisdiction. (Inserted map shows Bratten’s location in the Skagerrak). Source of data: The Swedish Agency for Marine and Water Management, Gothenburg, Sweden

The conflict between conservation and the fishery interests is genuine and can only be understood based on information about the degree of the encroachment on the specific fishery involved, including costs (Fig. 2). These controversies often arise from requests by conservation interests for regulation or even banning of fishing in an MPA (Ruiz-Frau et al. 2015; Horta e Costa et al. 2016). Conservation controversies with the fishery also occur when certain protected species, e.g. seals (*Phocidae*), interfere with the mainly small-scale coastal fishing.

#### Traditional rights

The recognition of “traditional” fishing rights is a major issue in resource and environmental policy at both national and international levels (Dyspriani 2011; Grip 2017). In this respect, it is worth noting that property rights are absent or weak in the Sea, even though historical fishing rights are recognized in UNCLOS (Bernard 2012). Restrictions on traditional fishing in a conservation MPA often cause conflict, and potential compensation for lost fishing does not automatically follow from infringements on property rights, as on land. This often means that no compensation is given for lost fishing, aggravating the conflicts between the two interests.

### Environmental assessments in fisheries

Fisheries change the structure and functioning of marine ecosystems. To provide relevant information on the environmental effects of a particular fishery, use of SEA and EIA has been proposed to supplement current used tools for fishery assessment and the MSP process (CONSSO 2006; Brown and Hjerp 2006).

In oceans and seas, there is often a dearth of knowledge on the trends and impact of the main fisheries. While the targeted resources and ecosystem effects are well documented for some fisheries, for others knowledge of ecosystem structures and functions and exploited stocks is limited. Also, there is growing awareness of the linkages between sustainable fishery development and marine conservation. In that respect, SEA for plans and EIAs for projects can improve information on the ecosystem effects of a fishery.

The aim of SEA for a particular fishery is to consider its environmental effects early in the planning and decision-making process. SEA could be developed for strategic fishery plans in industrial fishing, e.g. sand eel (*Gymnammodytes* or *Ammodytes* spp.) and deep-sea fishing (Mac Donald 2008).

The aim of EIA for a fishery is to create conditions for better consideration of the environmental effects of particular fisheries (Dayton et al. 1995; Bowden and Leduc 2017). A systematic use of EIA in fisheries management could be developed, e.g. for evaluating the environmental consequences of introducing new fishing methods or important changes in current methods (CONSSO 2006). Of course, an introduction of SEA and EIA would have costs that would need to be considered in the socio-economic assessment of the activity (Box 3).

**Table:** 

**Box 3.** Resistance to the use of SEA and EIA in fisheries	
The fisheries sector has strongly opposed the introduction of SEA and EIA in fisheries. Among the problems listed are (Brown and Hjerp 2006): - who should carry out the assessments, - who should pay and - how will potential conflicts be resolved? From the preparatory work for the Swedish Environmental Code in the 1990s, it appears that the use of EIA within the fisheries was discussed, but in the end, it was considered that a fisheries EIA was not the same as an EIA under the Environmental Code, and the term was replaced by the analysis of the impact of fishing methods (20§ Sect. 2, Swedish Fisheries Act). The reason was uncertainty as to how sectoral laws such as the Fisheries Act (under Ministry of Agriculture) and the Environmental Code (under Ministry of Environment) should be coordinated and about who has the responsibility for monitoring and control (Christiernsson and Michanek 2016). Also, improved coordination would require legislative changes.	

There is an EU directive on EIA (Directive 85/337/EEC as amended 1997, 2003 and 2009), but the Directive is primarily aimed at activities that are more site-oriented than fisheries. Annex II to the Directive mentions activities similar to fisheries, for example agriculture and forestry. Considering this, it should be possible to apply EIA also to fishing, with certain modifications.

## The Marine Spatial Planning process

**Fig. 3:**
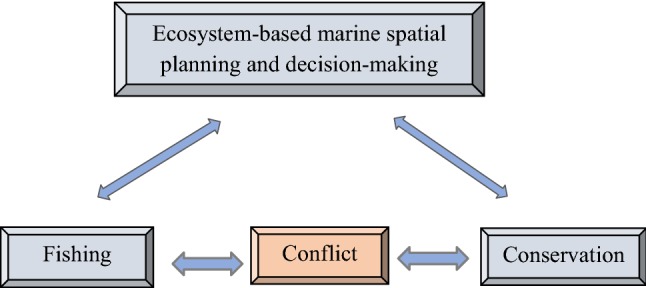
Conceptual outline of conservation conflicts with fishery or any other conflicting interest; from an ecosystem-based marine spatial planning and decision-making process (Crowder and Norse 2008). This process should not be tied to a specific sector, but to a national body with an overall planning responsibility

Marine spatial planning is about determining how water areas are to be used and how different public interests are to be weighed against each other in an open and democratic process, while considering the rights of individuals and other concerned interests (Fig. 3).^3^ Also, as planning is linked to policy-making, the process must involve stakeholders, planners and decision-makers. The planners provide scientific-based information that bears on decisions concerning the policy of the problem to be solved (Faludi 1973).

### The MSP procedure

MSP has become an important decision-support instrument for sectoral coordination and balancing different usages and regulations of sea resources, including marine nature conservation and industries such as fishery (Flannery and Ellis 2016). The MSP process includes consideration to both socio-economic impacts and ecological effects on the marine environment, and its biodiversity and ecosystem services (Crowder and Norse 2008). The EU has encoded its Maritime Policy (EU/EC 2007) in the Framework Directive for maritime spatial planning (Directive 2014/89/EU), and UNESCO/IOC has also developed a general guide to marine spatial planning (Ehler and Douvere 2009).

MSP involves negotiations between parties, supported by their often strong sectoral legislation. Currently, the dominating MSP approach is sectoral (Jones et al. 2016). Usually, each sector eagerly defends its mandate which may hamper the needed dialogue. A well-developed broader MSP instrument, not bound to a specific sector, can more easily bring concerned parties to the negotiation table to discuss opportunities for coordination of the interests involved and how to find a compromise. However, all negotiations of this kind have political implications and the dialogue between the parties does not always work.

Managers (planners) need to balance protection with sustainable use and supplement their knowledge through dialogue with a range of stakeholders (non-governmental environmental organizations and organizations representing users and exploiters). Also, some issues have cross-boundary effects, and even international implications at the political level, related to international agreements and the work of regional marine environmental and fishery organizations (Johannesen and Lassen 2014; UN Environment 2018).

Various Regional Commissions for Fisheries of the Food and Agriculture Organization (FAO) of the United Nations (UN) have recently promoted the application of MSP to fisheries (FAO 2016b). However, this process is focused on achieving specific sectoral objectives and is a form of adaptive planning related to national fishing priorities (Jones et al. 2016).

#### Differences in planning

Critical scientific studies and careful analyses are crucial as support for responsible planning.^4^ Usually, the planning leads to the formulation of different types of plans providing an independent overview and a grading or prioritization between different user interests. In the planning process, it is the planner who draws up a plan or alternative plans on possible ways forward. However, as stressed by Faludi (1973), “the final decision is taken by the decision-maker, not the planner”.

As different forms of planning influence each other, planning is considered as a general approach to decision-making and is not tied to the activities of any profession or department of government (Faludi 1973). The current sectoral planning and management related to nature conservation and fishery should become more inter-sectoral to ensure that marine environmental governance and management in Sweden, and elsewhere complies with the needs of society. This requires consideration of ecosystem functions, relevant socio-economic activities and the interests of the public in an overall spatial planning and decision-making process. It also means that the results of both the natural science and social sciences need to be integrated and considered in the MSP process (Foley et al. 2010; Ruiz-Frau et al. 2015; Gall and Rodwell 2016).

It should be noted that MSP practices differ between countries depending on their administrative, jurisdictional and cultural rules and traditions. Also, while the incentives for using a broader MSP process are growing, they are still rather weak.

## Discussion

Around the world, conservation claims are leading to controversies with the fishery (Montevecchi 2001; Salomon et al. 2011; Pendleton et al. 2017). As an example, the maritime planning and decision-making process within the EU regarding MPAs for conservation purposes is laden with problems and the discussion of fishing and conservation interests repeatedly takes place in separate fora (Johannesen and Lassen 2014). Usually, the fishery is managed separately from the environment, both nationally and internationally, and there is often a glaring lack of dialogue and cooperation between the two interests.

The interactions between conservation MPAs and fisheries resource management are complex, with different goals and different management agencies, (e.g. states, local communities, companies, trusts and others) responsible for implementing these goals. Conservation MPAs are created for long-term conservation, while fisheries management agencies typically have a mandate to maximize fisheries yield. An overall MSP instrument can facilitate and improve the negotiation process of the parties, but it has no guarantee for solving conflicts.

Naturally, the administrative and legal systems of a country affect how the management of nature conservation and fishing interests are handled and implemented by responsible agencies. However, most countries follow common principles for how laws and regulations are used (Pomeroy et al. 2005). With regard to implementation, there are differences between countries, especially when it comes to enforcement and control of MPAs (Halpern 2014; Grorud-Colvert et al. 2019).

It should be noted that the fishery has a long and strong tradition in most coastal countries, while the need for marine nature conservation was basically first given prominence by the 1992 UN Conference on Environment and Development (Chapter 17 of Agenda 21). Today, the interest in marine nature conservation and the need for establishing MPAs are increasing, and thus also there are the potential conflicts with fishery. The current conservation trend is to establish bigger, sometimes very big, MPAs in order to reach the UN Convention on Biodiversity and Aichi target 11 and the UN Sustainable Development Goal 14.5 of 10% of the ocean area protected by 2020 (Grorud-Colvert et al. 2019). This in turn has often led to a strengthening of the legislation for managing conflicts between nature conservation and other interests, including commercial fisheries in the countries concerned.

The different legal and administrative principles that apply to the planning, establishment and management of MPAs for nature conservation and fishery purposes need to be observed to properly handle these conflicts (Klein et al. 2008; Salomon et al. 2011). Also, the purpose of an MPA needs to be defined in measurable terms so that the outcome can be assessed.

*Weaknesses in planning*

All planning has its limitations and deficiencies. The planning of future resource usage has become bureaucratic and often distant from those most affected. Public planning is primarily determined by and adapted to economic demands. As an example, there is a growing concern within the EU about the tensions between the Marine Strategy Framework Directive (MSFD) and the Directive for Maritime Spatial Planning. This concerns how the MSP process can combine sustainable maritime growth (e.g. for fisheries) according to the Maritime Policy with Good Environmental Status according to the MSFD, for instance, for the conservation of marine biodiversity (Jones et al. 2016).

The problems and deficiencies encountered when using MSP, e.g. related to fishery and ecological resources, have been addressed in recent contributions (Cicin-Sain and Belfioreb 2005; Metcalfe et al. 2015; Flannery and Ellis 2016; Janßen et al. 2018). Janßen et al. (2018) reviewed the practical problems that occur when fishing interests are involved in the MSP process, e.g. concerning where fishermen fish, seasonal dynamics and how to deal with spatial patterns and ecological processes. In our view, these challenges in fisheries management require the broader information that can be provided by EIAs.

These challenges are not new and can be surmounted (Ackefors and Grip 1995; Johannesen and Lassen 2014; FAO 2016b). In an example from Sweden, a comprehensive evaluation method (based on biological, chemical, physical and economic criteria) to manage fishery interests in the MSP process was developed in the 1980s (Grip 1992).

Flannery and Ellis (2016) argue for an MSP process with, among other things, a more equity-based democratic decision-making and a fairer distribution of marine resources. It is essential to understand that social, cultural, economic and political attributes overlay the biological and ecological aspects. These aspects can be met in a multisectoral marine spatial planning and decision-making process. In this process, all concerned stakeholders and the public should participate, e.g. regarding the conflict between the usage of marine fish resources and claims related to the establishment of MPAs for conservation purposes (Reed 2008; Agardy 2010; Gall and Rodwell 2016; Hassler et al. 2018; Rabe 2018).

Looking ahead, we see four essential aspects (a–d) that could improve management of the conflicts:

### (a) Marine spatial planning and conflicting interests

The sustainable use and protection of marine environments and their resources requires integrative planning and management practices. To achieve a balanced weighing of fishery and conservation interests in a proposed MPA, the relevant fishery and nature conservation objectives must be properly identified (Douvere 2008; Douvere and Ehler 2009; Carneiro 2013).

The appropriate instrument for making such coordination possible and determining which demands on water resources should be prioritized has, despite remaining deficiencies, been proven to be an overall, multisectoral and ecosystem-based marine spatial planning and decision-making process (Browman et al. 2004). This process, which today is promoted globally, should not be bound to a specific sector and should consistently distinguish between the establishment of MPAs for nature conservation and for fisheries resource management.

The process should involve all relevant stakeholders and use an ecosystem-based approach to the use of fish resources that relates to the stock’s productive capacity and considers the environmental effects of the fishing (Crowder and Norse 2008; Foley et al. 2010; Guerry et al. 2012). It should also make clear which national body has the overall mandate to make the final decision and implement the planned activities. Otherwise, sector-bound planning and decision-making will continue to create management problems.

Also, cross-boundary effects related to international agreements and implications at the political level need to be taken into account. Furthermore, a multisectoral and ecosystem-based MSP process should, with some complements, be applicable also in the High Seas, including the seabed, provided that the lack of regulation under the UN Convention on Law of the Sea is addressed (Ardron et al. 2008).

### (b) SEA and EIA in fisheries

So far, SEA and EIA are not commonly used tools for assessing the environmental impacts of capture fisheries, although the issue, including cost implications, has been addressed (Brown and Hjerp 2006; CONSSO 2006; Hobday et al. 2011). This is strange. The diversity of fisheries and their environmental effects are a particularly strong argument for introduction of situation-specific SEA and EIA in many fisheries where there is a lack of knowledge. These instruments should be used and paid for in the same way as for other activities and to supplement more commonly used fisheries assessment and management tools. The focus should be on the direct and indirect effects of the relevant fisheries on marine ecosystems. The scientific competence is there, but the costs have so far not been accepted.

SEA and EIA would provide highly relevant information on the environmental effects of fisheries, for instance, SEA on strategic fishery plans and EIA for certain fisheries, e.g. effects on birds of gillnet fishing (Thompson et al. 1998; Žydelis et al. 2013), long line fisheries (Anderson et al. 2011) and other fishing nets (Strann et al. 1991) and on dolphin by-catch (Snape et al. 2018). Improved information on the direct and indirect ecosystem effects of relevant fisheries would support the MSP and decision-making in conflicts between fisheries and marine nature conservation.

### (c) Distinguish between MPAs for conservation and fishery

In our review, we have found that there is often confusion about the aims of MPAs for nature conservation and fishery’s resource management. MPAs are useful tools for both marine nature conservation and fisheries resource management. However, their objectives are different, and one should distinguish between them in the MSP and decision-making processes depending on whether the MPA is established for conservation of ecosystems and biodiversity or for fisheries resource management.

We argue that the practical management of fishing in an MPA for nature conservation purposes (e.g. a nature reserve) should be regulated and managed under the conservation law, just as other activities. Correspondingly, a fishery MPA for resource management (e.g. a fishery no-take MPA) should be managed and regulated according to the fishery law. Management of conservation MPAs according to two different sectoral laws invites target conflicts and should be avoided (Enderby and Enderby 2006; Dudley 2008; Johannesen and Lassen 2014).

### (d) Fully protected conservation MPAs

Finally, we argue that MPAs for nature conservation in the highest protection category should be established as marine national parks and marine nature reserves. Vulnerable environments protected as conservation MPAs in the highest protection category need stricter protection and should be fully protected from activities that have negative effects on the habitats and species you want to protect. This is an issue related to political will and should be legally supported. Only a few coastal marine countries have a conservation legislation that allows the establishment of fully protected marine nature reserves (Costello 2018). However, we argue that conservation MPAs in the highest protection category (marine nature reserves and national parks) need to be strictly protected in order to fill the purpose for which they were created.

Strictly protected marine nature reserves, as in New Zealand, benefit the environment and biodiversity and should be promoted (Fogarty and Murawski 2005; Ballantine 2014; Costello 2018). Their regulation should apply to all activities and should facilitate the management of established fully protected MPAs. Such full protection would strengthen public confidence in nature conservation and be beneficial to fishery, also outside the MPAs (Costello 2018).

## Acronyms

CONSSO: Conference on North Sea Senior Officials
EIA: Environmental Impact Assessment
EU: European Union
FAO: Food and Agriculture Organization of the UN
HELCOM: The Helsinki Commission for the Helsinki Convention on the Protection of the Marine environment in the Baltic Sea Area
IOC: Intergovernmental Oceanographic Commission
MPA: Marine Protected Area
MSC: Marine Stewardship Council
MSFD: Marine Strategy Framework Directive
MSP: Marine Spatial Planning
OSPAR: The OSPAR Convention for the Protection of the Marine Environment of the North-East Atlantic
SEA: Strategic Environmental Assessment
UN: United Nations
UNEP: United Nations Environment Programme
UNESCO: UN Educational, Scientific and Cultural Organization

## Footnotes

1 The first steps in developing an ecological or ecosystem-based approach to marine spatial planning were taken early by Sweden (Carlberg and Grip 1982). It was based on the principle that prior to decisions that cause an intrusion on the environment, the environmental effects of the decision, as well as the current environmental status of the affected land or water area should be known.

2 A change may be under way. In December 2017, the General Assembly decided to convene an Intergovernmental Conference on an international legally binding instrument under the United Nations Convention on the Law of the Sea. The conference deals with the conservation and sustainable use of marine biological diversity of areas beyond national jurisdiction (General Assembly resolution 72/249). The conference will meet in four sessions. In September 2018, the first session was held. The second and third sessions will be convened in 2019, and the fourth session in the first half of 2020.

3 MSP is commonly defined as a process of public authorities for analysing and allocating the spatial and temporal distribution of human activities in marine areas, to achieve ecological, economic and social objectives. It is an instrument for reducing conflicts and strengthening the coordination between sectors and for protecting the environment by taking the environmental effects of considered activities into account (European Union 2011).

4 This has long been recognized (Beer 1966; Quade 1968; Faludi 1973; Guerry et al. 2012). According to Quade (1968), it means that conscious efforts are made to increase the validity of policies by which human beings transform their environment.
